# Tertiary alcohol preferred: Hydroxylation of *trans*-3-methyl-L-proline with proline hydroxylases

**DOI:** 10.3762/bjoc.7.193

**Published:** 2011-12-05

**Authors:** Christian Klein, Wolfgang Hüttel

**Affiliations:** 1 Institute of Pharmaceutical Sciences, Department of Pharmaceutical and Medicinal Chemistry, Albert-Ludwigs-Universität Freiburg, Albertstr. 25, 79104 Freiburg, Germany

**Keywords:** asymmetric catalysis, enzyme catalysis, hydroxyproline, α-ketoglutarate dependent iron(II) oxygenases, regioselectivity, stereoselectivity

## Abstract

The enzymatic synthesis of tertiary alcohols by the stereospecific oxidation of tertiary alkyl centers is a most-straightforward but challenging approach, since these positions are sterically hindered. In contrast to P450-monooxygenases, there is little known about the potential of non-heme iron(II) oxygenases to catalyze such reactions. We have studied the hydroxylation of *trans*-3-methyl-L-proline with the α-ketoglutarate (α-KG) dependent oxygenases, *cis*-3-proline hydroxylase type II and *cis*-4-proline hydroxylase (*cis*-P3H_II and *cis*-P4H). With *cis*-P3H_II, the tertiary alcohol product (3*R*)-3-hydroxy-3-methyl-L-proline was obtained exclusively but in reduced yield (~7%) compared to the native substrate L-proline. For *cis*-P4H, a complete shift in regioselectivity from C-4 to C-3 was observed so that the same product as with *cis*-P3H_II was obtained. Moreover, the yields were at least as good as in control reactions with L-proline (~110% relative yield). This result demonstrates a remarkable potential of non-heme iron(II) oxygenases to oxidize substrates selectively at sterically hindered positions.

## Findings

Enantiomerically pure tertiary alcohols are valuable building blocks for the synthesis of natural products, biologically active compounds, and pharmaceuticals. However, their stereoselective synthesis is often challenging, as the reaction centers are sterically hindered or electronically disfavored. In addition to numerous approaches for the synthesis of tertiary alcohols with classical organic chemistry [1–5], enzyme-catalyzed approaches have also been successfully established, and especially, hydrolases, i.e., lipases and esterases, are used for the kinetic resolution of tertiary alcohols [6–9]. Other less common approaches include stereospecific enzyme-catalyzed hydrolyses of epoxides, stereoselective additions to ketones with hydroxynitrile lyases or carboligases, and the application of enzymes involved in terpene biosynthesis, such as dehydratases, cyclases or transferases [6,8]. An approach whose potential has not yet been fully exploited is the stereospecific hydroxylation of tertiary alkyl moieties with oxygenases. Most oxidations to tertiary alcohols described so far were observed during degradation of steroids and other terpenoid bioactive compounds by microbial whole cells [10–12]. Hydroxylations to tertiary alcohols with isolated or heterologously expressed enzymes have mostly exploited P450-monooxygenases. The application of these enzymes for chemical synthesis has been recently reviewed in several articles [13–16].

**Scheme 1:**
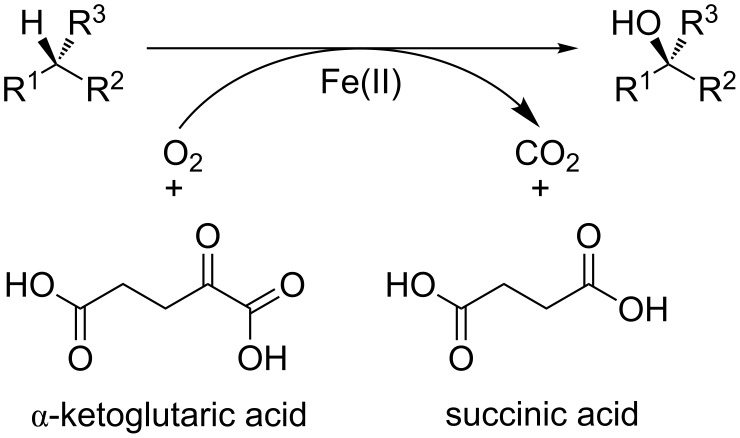
Catalytic cycle of α-KG dependent oxygenases.

In contrast, little is known about the ability of non-heme iron(II) enzymes to oxidize tertiary carbon centers. To our knowledge, the formation of tertiary alcohols with α-ketoglutarate (α-KG) dependent iron(II) oxygenases has not been previously reported. These enzymes typically catalyze CH-activation reactions in primary and secondary metabolism [17–21]. For the catalytic cycle, one α-KG and one oxygen molecule are required, besides the main substrate. The ketoacid is decarboxylated oxidatively by one oxygen atom from O_2_, whereas the other is used for substrate oxidation (Scheme 1).

Here, we describe a regio- and stereospecific hydroxylation of *trans*-3-methyl-L-proline to (3*R*)-3-hydroxy-3-methyl-L-proline with two different proline hydroxylases.

**Scheme 2:**
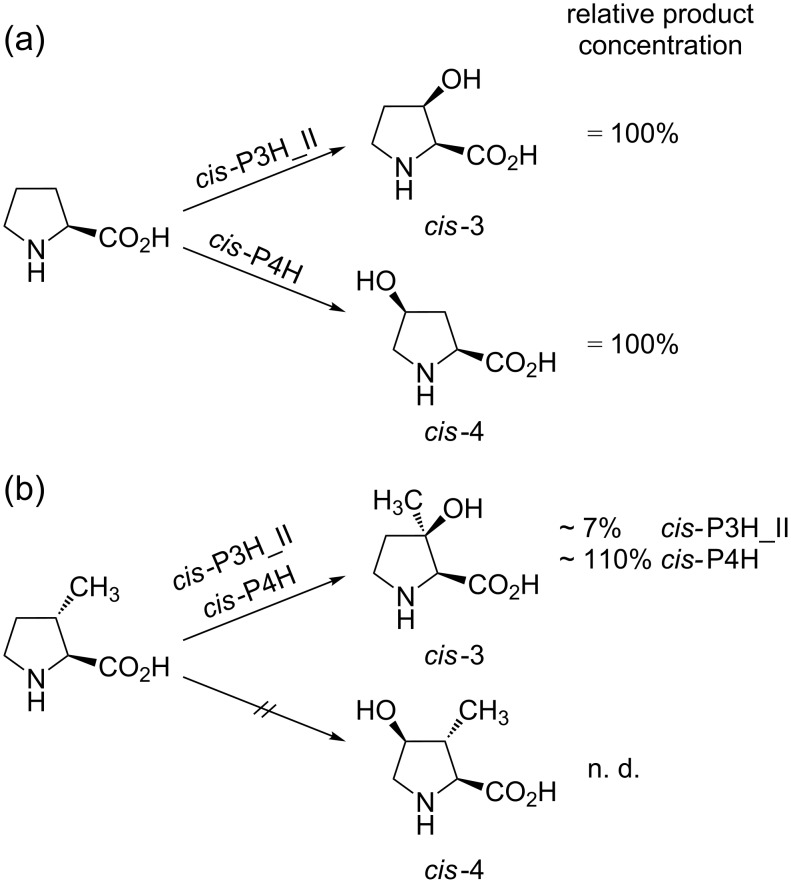
Selectivities and relative yields in conversions of (a) L-proline (defined as 100% yield) and (b) *trans*-3-methyl-L-proline with *cis*-P3H_II and *cis*-P4H.

In contrast to the mechanistically related and more common prolyl hydroxylases, which accept peptide bound proline as a substrate and play a key role in collagen biosynthesis, proline hydroxylases exclusively hydroxylate the free L-amino acid and are limited to some bacteria and filamentous fungi. As far as it is known, they are involved in secondary metabolism, for example, in the biosynthesis of the non-ribosomal peptide antibiotics etamycin, telomycin and pneumocandin [22–24]. So far, five bacterial proline hydroxylases have been cloned and overexpressed in *E. coli*: A *trans*-4-proline hydroxylase (*trans*-P4H) from *Dactylosporangium* sp. [25] two *cis*-3-proline hydroxylase isoenzymes from *Streptomyces* sp. strain TH1 (*cis*-P3H_I and *cis*-P3H_II) [26–27] and two *cis*-4-proline hydroxylases (*cis-*P4H) from *Sinorhizobium meliloti* and *Mesorhizobium loti* [28]. Since hydroxyprolines are important chiral building blocks for chemical synthesis [29–30], a procedure for the large-scale production of *cis*-3- and *trans*-4-hydroxyproline was established in which a recombinant *E. coli* strain expresses the corresponding proline hydroxylase [31–33]. Recently, we presented an analogous approach for synthesis on a laboratory scale in combination with a significantly simplified method for product purification [34]. This allows the production not only of hydroxyprolines, but also of hydroxylated proline derivatives on a preparative scale. This system provides an ideal platform for further studies on proline hydroxylase activities with new substrates or enzyme variants. Previous testing showed that the substrate specificity of the enzymes is strict towards the secondary amino acid moiety, but “relaxed” towards changes in the carbohydrate backbone of L-proline [35]. We therefore incubated the commercially available *trans*-3-methyl-L-proline for 16 h with purified *cis*-P3H_II and *cis*-P4H (*Sinorhizobium meliloti)*, which hydroxylate the natural substrate L-proline to *cis*-3- and *cis*-4-hydroxyproline, respectively (Scheme 2).

**Figure 1:**
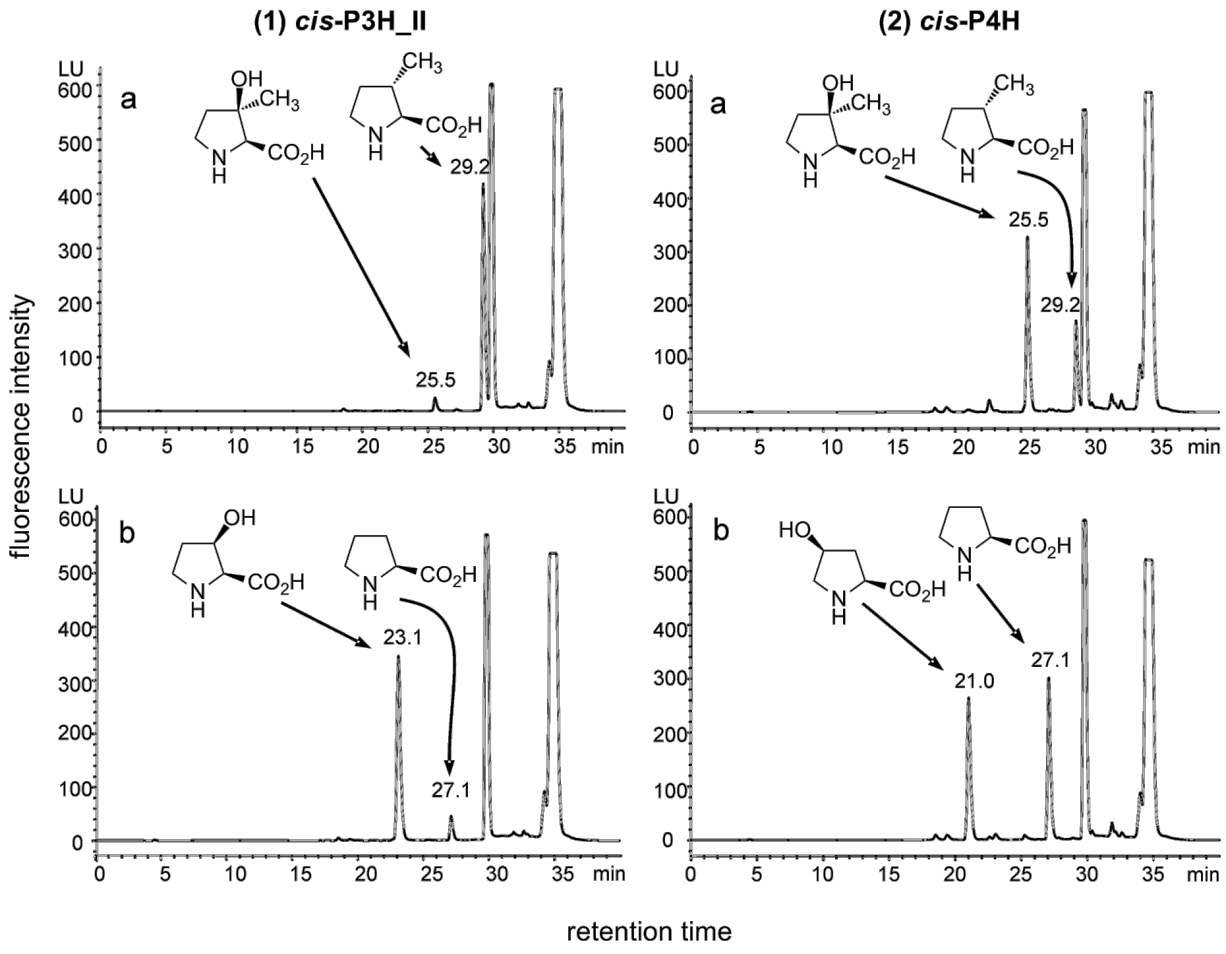
Typical HPLC-chromatograms of the conversions of (a) *trans*-3-methyl-L-proline and (b) L-proline under identical conditions with (1) *cis*-P3H_II and (2) *cis*-P4H. Yields are dependent on the amount of enzyme used. In this example the quantities are: (1)(a): 6.4% (0.25 mM); (1)(b): 90% (3.6 mM); (2)(a): 67% (2.7 mM); (2)(b): 50% (2.0 mM). The more significant relative yields in comparison to a reference reaction with L-proline are discussed in the text. Peaks at retention times >30 min are due to Fmoc-adducts formed during the derivatization reaction. Fluorescence assay wavelengths: Excitation = 254 nm, emission = 316 nm.

For reference, L-proline was converted in parallel with an identical amount of the enzyme preparation. The samples were then analyzed by HPLC by using a fluorescence assay [34]. Since the fluorescence activity of the Fmoc-derivatized proline and derivatives that we have investigated is virtually identical (data not shown), and the measured emissions were in a linear range, the peak areas were used for an approximate quantification of the compound concentrations (Figure 1). In case of *cis*-P3H_II, a new compound was found, but product concentrations were only approx. 7% compared to those obtained in conversions of L-proline. Based on the *cis*-3-selectivity of the enzyme we assumed that the tertiary alcohol was formed. Surprisingly, the conversion with *cis*-P4H gave a product with the same retention time as the product of *cis*-P3H_II, but in much better relative yield (~110% compared to the L-proline control) (Figure 1a).

To determine their structure, the products were produced on a semi*-*preparative scale; 26 mg *trans-*3-methyl-L-proline was converted in vivo in 100 mL *E. coli* cultures, overexpressing the corresponding enzyme [34]. After full conversion, the supernatants were purified by ion-exchange chromatography and the products were analyzed by one- and two-dimensional NMR techniques (see NMR and MS spectra in Supporting Information File 1). It was found that both enzymes indeed yielded the same compound, which was identified unambiguously as the C-3 hydroxylated product.

Since the substrate has L-configuration and the enzyme is strictly *cis*-diastereoselective, it can be assumed that (3*R*)-3-hydroxy-3-methyl-L-proline is the product. 1D- and 2D-NOESY NMR-spectra clearly show a correlation between the methyl group and the proton at C-2, suggesting a *cis* position for these substituents and, consequently, a *cis*-configuration for the tertiary alcohol (Supporting Information File 1). Whereas the reactivity of *cis*-P3H_II could be expected, the shift in regioselectivity and the high activity of *cis*-P4H is remarkable. A certain degree of flexibility in the regioselectivity of this enzyme was already found in conversions with the 6-ring-analogue of L-proline, i.e., L-pipecolic acid. In that case an approx. 1:1 mixture of the expected *cis*-5-hydroxypipecolic acid and its *cis*-3-isomer, which is also the product of *cis*-P3H_II, was obtained [34]. In general, it can be assumed that the shift of reactivity in the reaction with *trans*-3-methyl-L-proline is due to the increased stability of the tertiary radical intermediate at C-3 compared to the secondary at one C-4. However, this putative effect does not increase the reactivity of *cis*-P3H_II. So it is most likely that the complex interplay between kinetic and steric factors determines the reactivity of these enzymes. Further spectroscopic and structural data are required in order to provide an insight into the functionality of these enzymes. Nevertheless, our results show that α-KG dependent oxygenases have high potential for the production of tertiary alcohols. Both enzymes investigated afford only a single product selectively and, in the case of *cis*-P4H, the activity was comparable to that with the native substrate. Moreover, proline hydroxylases can be applied for whole cell biotransformations on a preparative scale. Even though the activity of the enzymes is still difficult to predict for conversions with unnatural substrates, highly efficient catalytic systems may be accessible from other α-KG dependent oxygenases.

## Supporting Information

File 1 Experimental section, analytical data (NMR and MS).
