# Supplementary material for: Tertiary alcohol preferred: Hydroxylation of trans-3-methyl-L-proline with proline hydroxylases
Source: Beilstein J Org Chem. 2011 Dec 5;7:1643–7. doi: 10.3762/bjoc.7.193 (PMC3252868; doi:10.3762/bjoc.7.193)
Supplement: File 1 — Experimental section, analytical data (NMR and MS). [file Beilstein_J_Org_Chem-07-1643-s001.pdf]

Supporting Information

for

Tertiary alcohol preferred: Hydroxylation of *trans*-3-methyl-L-proline with proline hydroxylases

Christian Klein and Wolfgang Hüttel*

Address: Institute of Pharmaceutical Sciences, Department of Pharmaceutical and Medicinal Chemistry, Albert-Ludwigs-Universität Freiburg, Albertstr. 25, 79104 Freiburg, Germany

Email: Wolfgang Hüttel - wolfgang.huettel@pharmazie.uni-freiburg.de

* Corresponding author

Email: Wolfgang Hüttel - wolfgang.huettel@pharmazie.uni-freiburg.de

* Corresponding author

Experimental section, analytical data (NMR and MS)

Table of contents

1. Experimental procedures S2

2. NMR-spectra of enzymatic conversions product S4

3. MS^2 spectrum of (3*R*)-3-hydroxy-3-methyl-L-proline S10

1) Experimental procedures

a) In vitro conversions

The enzymes *cis*-P3H_II and *cis*-P4H were produced in *E. coli* and purified as described before [S1]. The hydroxylation activities were measured in a reaction mixture containing Mes buffer (50 mM, pH 6.5), L-proline or *trans*-3-methyl-L-proline (4 mM), α-ketoglutarate (10 mM), ferrous sulfate (0.5 mM), L-ascorbate (1.5 mM) and enzyme preparation in a final volume of 1 mL. To compare enzymatic activities, identical amounts of the same enzyme preparation were used for the conversion of *trans*-3-methyl-L-proline and L-proline in parallel. Reactions with *cis*P3H_II were carried out at 37 °C and with *cis*-P4H at 25 °C for 16 h. Product formation was measured fluorometrically by HPLC at 254 nm excitation wavelength and at 316 nm emission wavelength after Fmoc-derivatization as previously described [S1]. Gradient program 2 [S1] was used with a LiChrosorb RP-18 (5 µ, 250 × 4.0 mm) or a LiChrosorb100 RP-18 EC (5 µ column, 250 × 4.0 mm) purchased form CS (Chromatographie-Service), Langerwehe, Germany. All retention times were verified with reference compounds.

b) In vivo production of (3*R*)-3-hydroxy-3-methyl-L-proline

100 mL of an amino-acid-free high-cell-density medium (Table S1) was used for cultivation of proline hydroxylase producing strains under aerobic conditions as described before for in vivo hydroxylation of L-pipecolic acid [S1]. At OD_{600} = 3–4 IPTG was added to achieve a final concentration of 0.2 mM. Subsequently, 26 mg of *trans*-3-methyl-L-proline (2 mM) and 14 mg $FeSO_4 \cdot 7H_2O$ (0.5 mM) were added. The cells were incubated for 72–120 h at 28 °C until conversions were complete. The reactions were monitored by HPLC. Ion-exchange chromatography was used for

isolation of (3*R*)-3-hydroxy-3-methyl-L-proline under the same conditions as described for hydroxyprolines [S1].

Table S1: High-cell-density medium [S2].

KH_2PO_4	16.6 g/L
NH_4NaHPO_4	4.0 g/L
citric acid	2.1 g/L
$Fe_2(SO_4)_3$	0.075 g/L
boric acid	3.8 mg/L
$MnCl_2 \cdot 4H_2O$	18.8 mg/L
$EDTA \cdot 2H_2O$	10.5 mg/L
$CuCl_2 \cdot 2H_2O$	1.9 mg/L
$Na_2MoO_4 \cdot 2H_2O$	3.1 mg/L
$CoCl_2 \cdot 6H_2O$	3.1 mg/L
$ZnSO4 \cdot 2H_2O$	10.0 mg/L
After autoclaving, addition of:	
glycerol	1% (v/v)
$MgSO_4 \cdot 2H_2O$	1.5 g/L
Adjust pH with 25% (v/v) NH_3 to	6.8

2) NMR spectra of enzymatic conversions product

(3*R*)-3-Hydroxy-3-methyl-L-proline = (2*S*,3*R*)-3-hydroxy-3-methylpyrrolidine-2-carboxylic acid

^{1}H NMR, ^{13}C NMR and ^{1}H^{1}H-COSY NMR, ^{1}H^{13}C HSQC NMR, 1D and 2D-NOESY

NMR spectra were recorded at 400 MHz (^{1}H) and 100 MHz (^{13}C) on a Bruker Avance DRX 400 (Buker Biospin GmbH, Rheinstetten, Germany) in D_2O or CD_3OD. Chemical shifts refer to H_2O (δ ^{1}H = 4.80 ppm) or methanol (δ ^{1}H = 3.34 ppm).

a) ^{1}H NMR

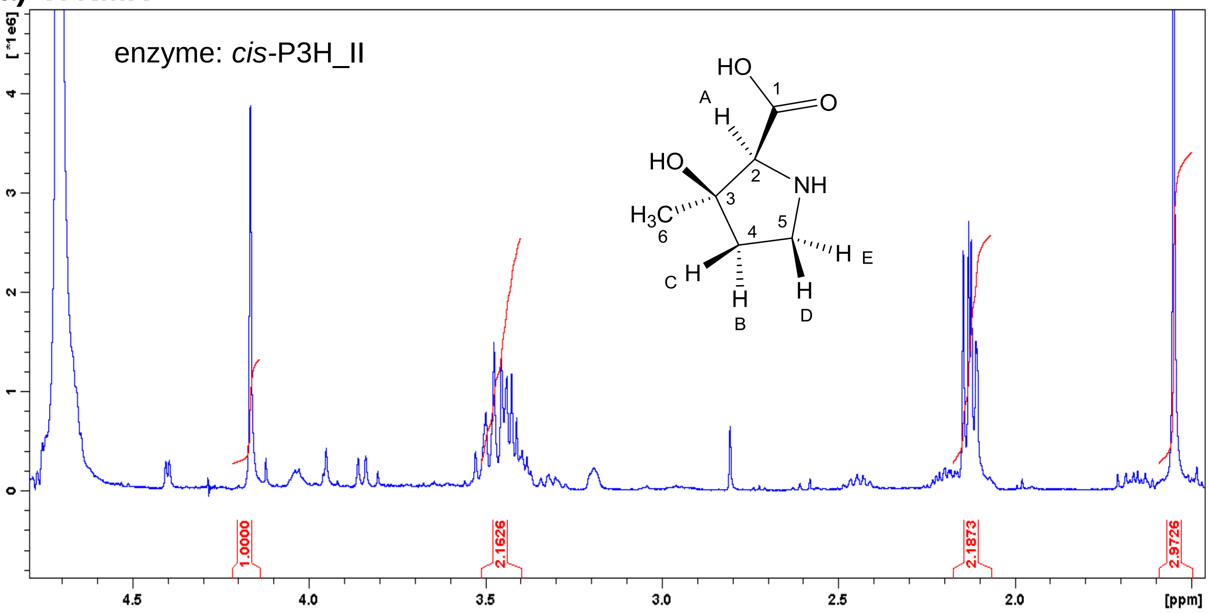

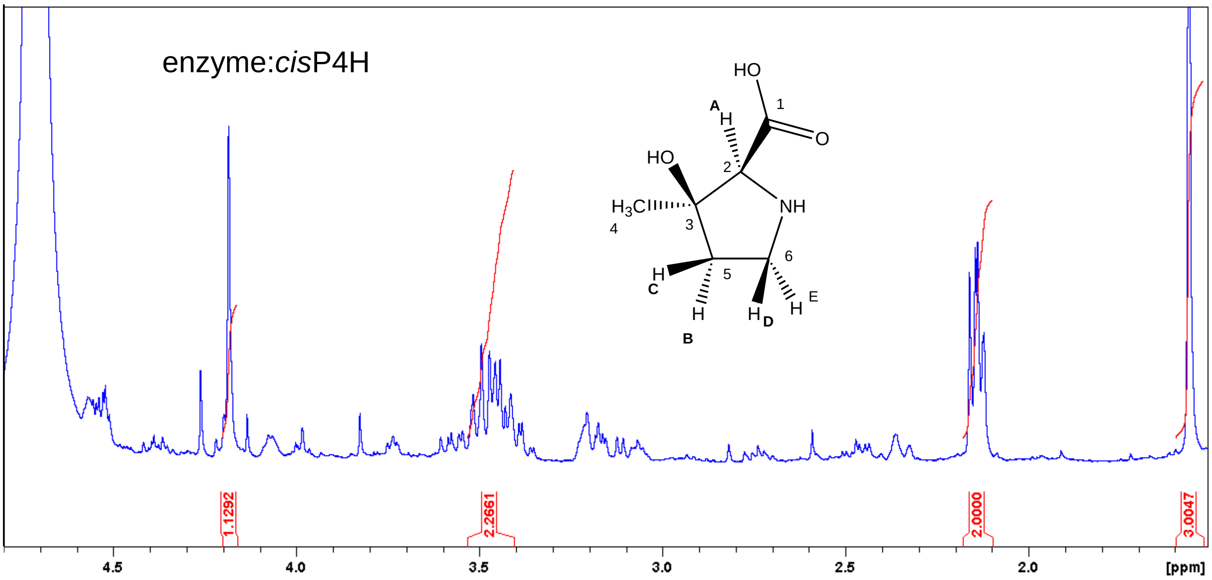

^{1}H NMR (400 MHz, D_2O) δ = 1.55 (s, 3H, C*H*$_3$), 2.13 (dd, *J* = 8.89 Hz, *J* = 6.10 Hz, 2H, C*H*$_2$-C, C*H*$_2$-B), 3.46 (m, 2H, C*H*$_2$-D, C*H*$_2$-E), 4.16 (s, 1H, C*H*-A).

b) ^{13}C NMR

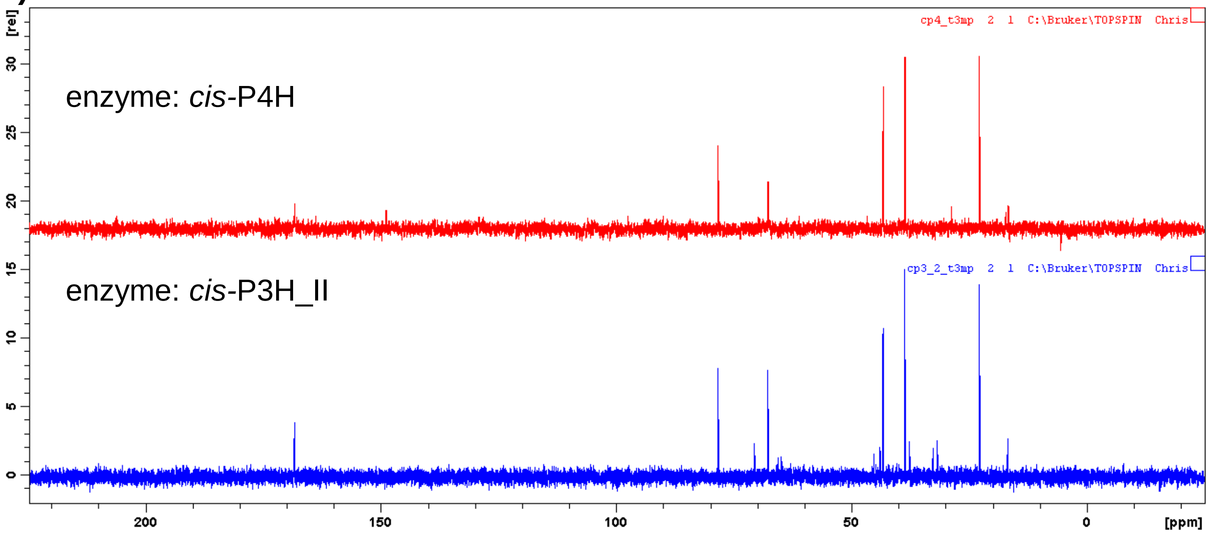

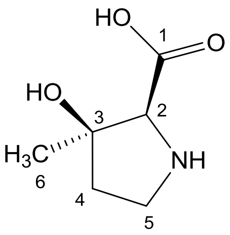

^{13}C NMR (100 MHz, D_2O) δ = 23.0 (C-6), 38.6 (C-4), 43.3 (C-5), 67.9 (C-2), 78.5 (C-3), 168.4 (C-1).

c) $^{1}H^{1}H$ COSY NMR

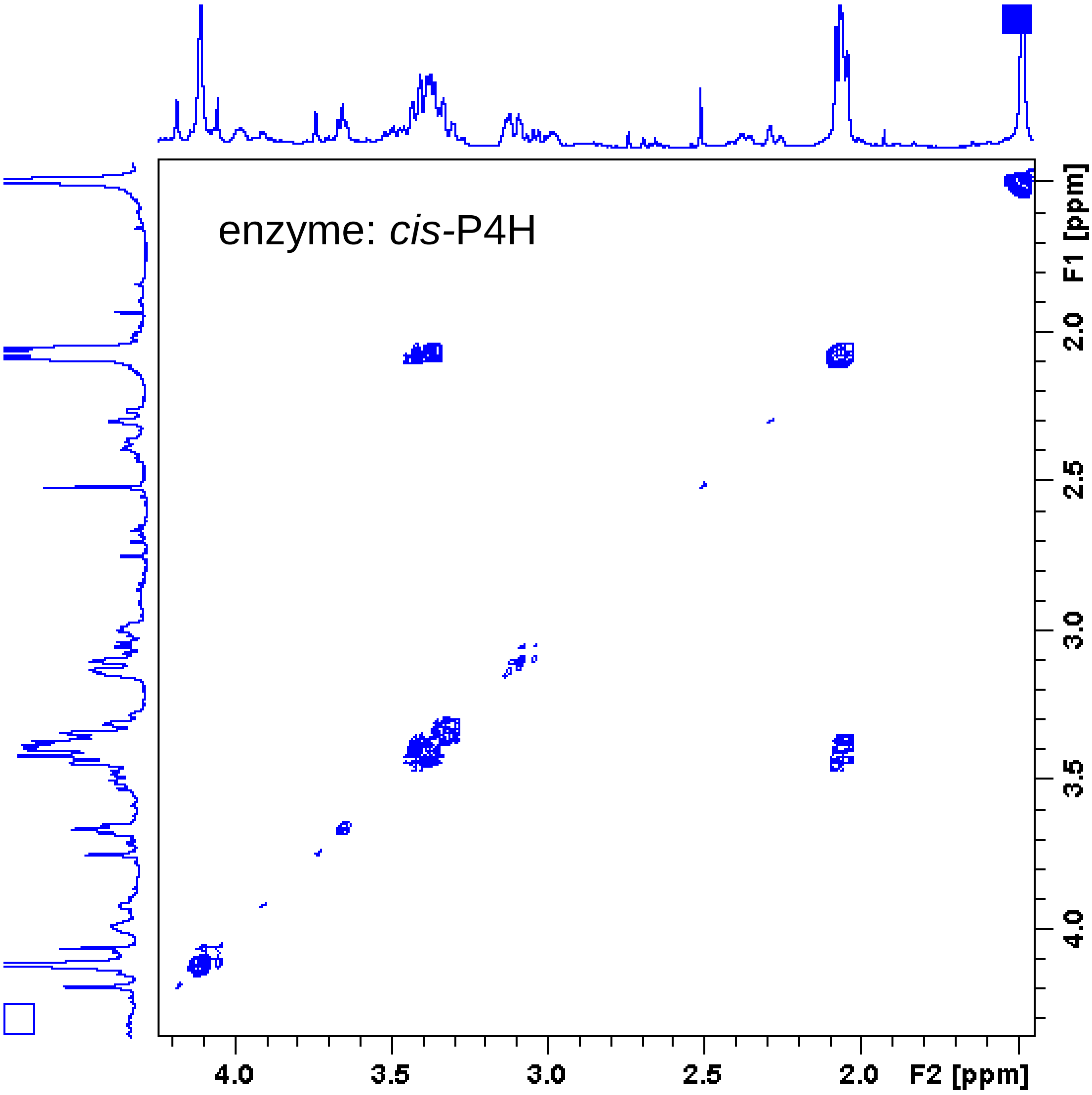

D_2O, 400 MHz, δ (ppm):

1.55	–
2.13	3.46
4.16	–

d) $^{1}H^{13}C$ HSQC NMR

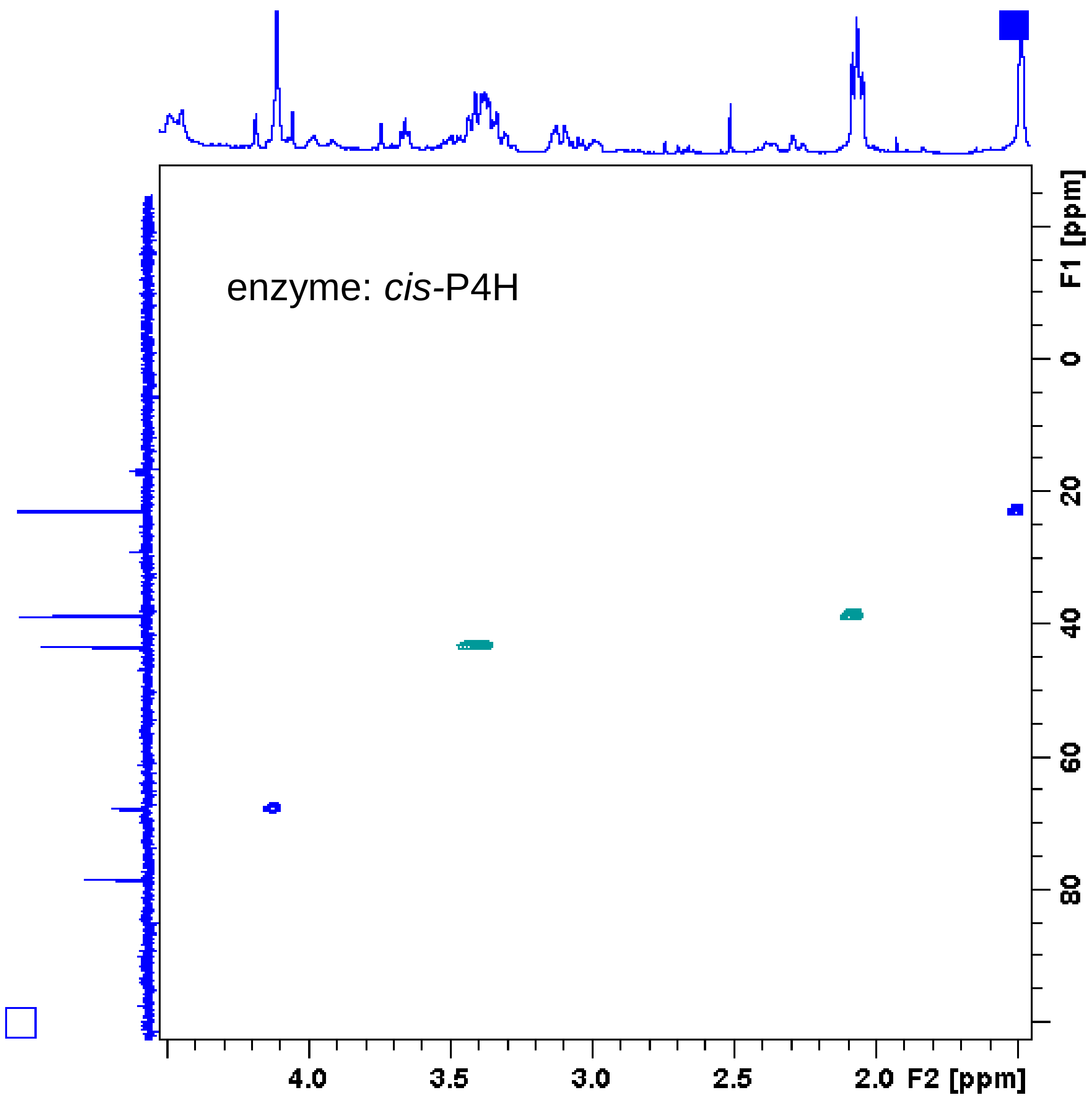

D_2O, ^{1}H: 400 MHz, ^{13}C 100 MHz, δ (ppm):

1.55	23.0	CH_3
2.13	38.6	CH_2
3.46	43.3	CH_2
4.16	67.9	CH

e) 2D-NOESY NMR

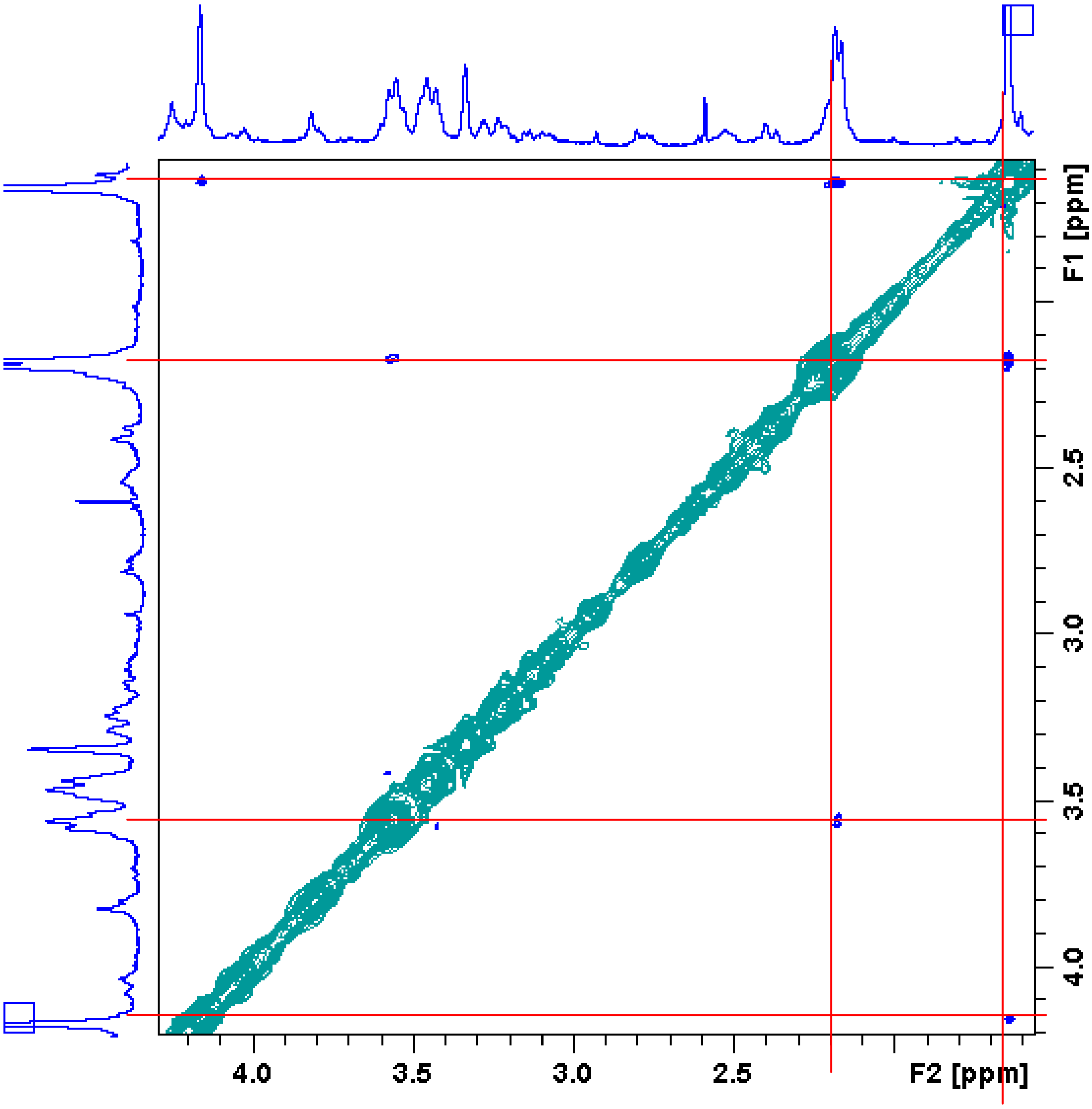

CD_3OD, (400 MHz)

1.65 ppm → 2.18 ppm; 4.16 ppm

2.18 ppm → 1.6 ppm; 3.56 ppm

f) 1D-NOESY NMR

irradiation at 1.65 ppm (CH_3)

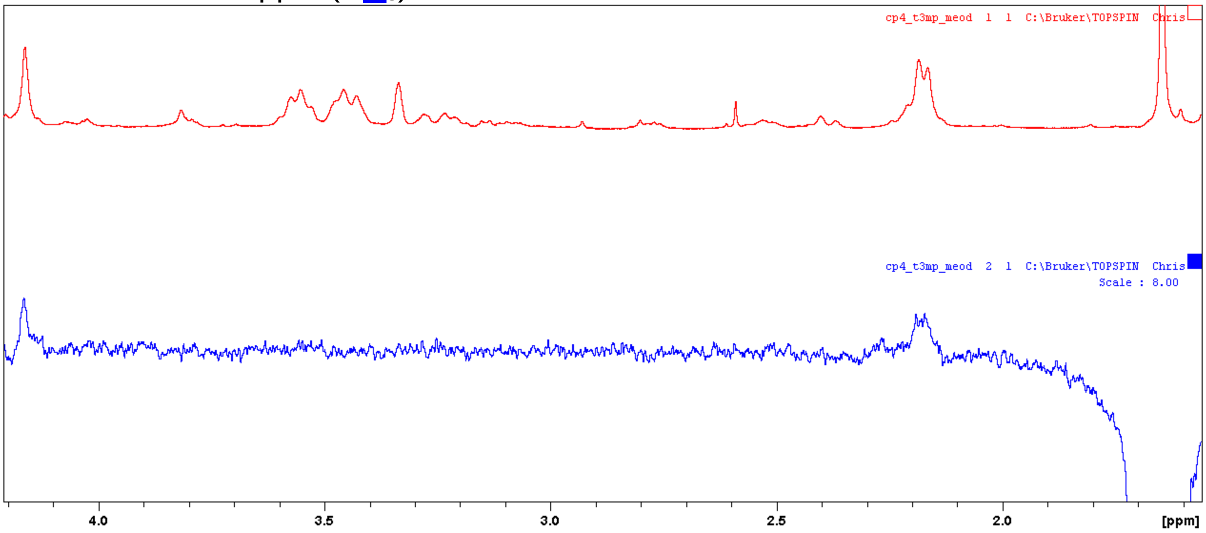

CD_3OD, (400 MHz)

1.65 ppm → 2.18 ppm; 4.16 ppm

irradiation at 4.16 (C*H*-A)

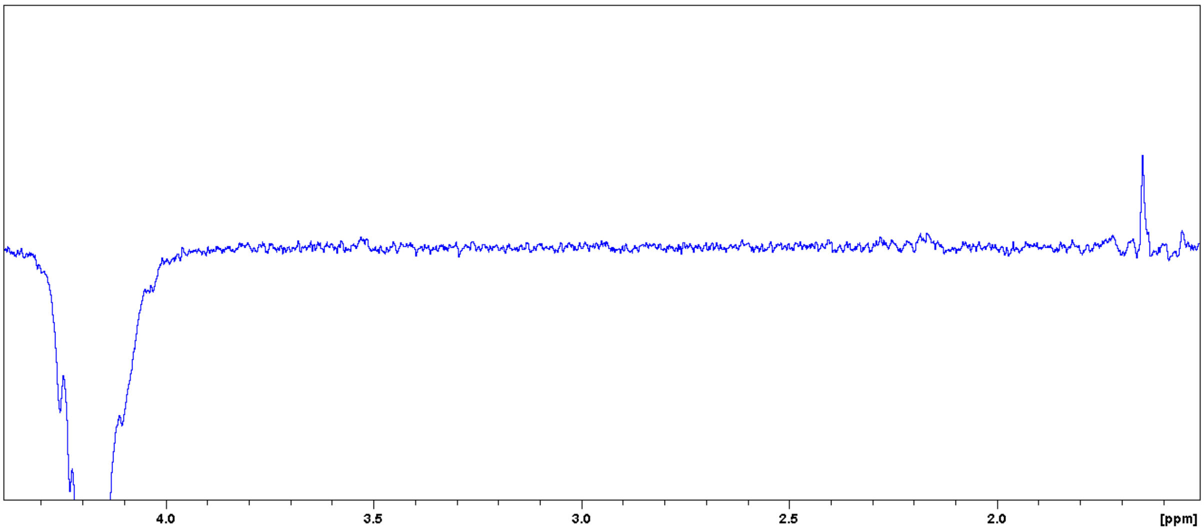

CD_3OD, (400 MHz)

4.16 ppm → 1.65 ppm

3) Mass spectrometry

MS- and MS^2 -analyses were performed with a API2000 Mass Spectrometer equipped with a turbospray ion source (Applied Biosystems, Carlsbad, CA, USA). Parameter: Product ion (MS^2): Ion source gas 1: 30 psi, ion source gas 2: 30 psi, collision gas: 6 psi, curtain gas: 10 psi, ionspray voltage: 5500 V, temperature: 400 °C, declustering potential: 50 V, focusing potential: 300 V, entrance potential: 10 V, collision energy: 25, collision cell exit potential: 3.

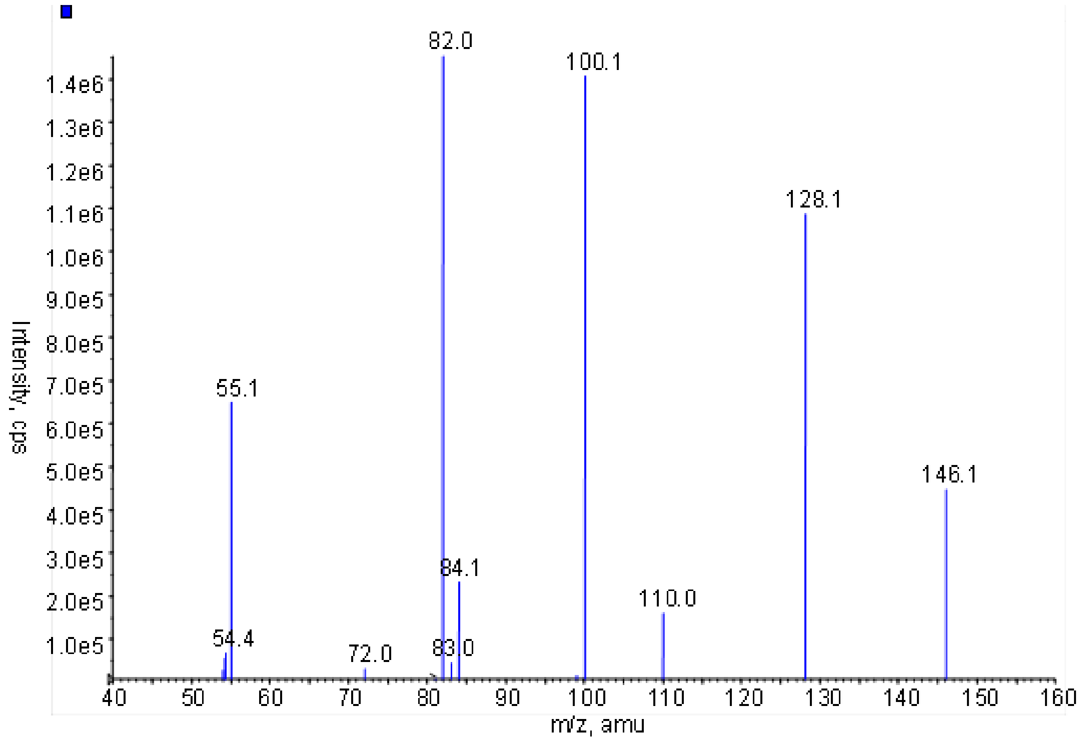

Tandem mass spectrometry: MS^2 of molecule peak 146 $[M + 1]^+$

MS^2 *m*/*z* (%) 146 (31) $[M + 1]^+$, 128 (75) $[C_6H_9NO_2 + 1]^+$, 110 (11) $[C_6H_7NO + 1]^+$, 100 (97) $[C_5H_9NO + 1]^+$, 84 (16) $[C_4H_5NO + 1]^+$, 82 (100) $[C_5H_7N + 1]^+$, 55 (45)

$C_6H_{11}NO_3$ MW: 145.16 g·mol^{-1}

References

S1 Klein, C.; Hüttel, W. *Adv. Synth. Catal.* **2011,** *353,* 1375–1383. doi:10.1002/adsc.201000863

S2 Horn, U.; Strittmatter, W.; Krebber, A.; Knüpfer, U.; Kujau, M.; Wenderoth, R.; Müller, K.; Matzku, S.; Plückthun, A.; Riesenberg, D. *Appl. Microbiol. Biotechnol.* **1996,** *46,* 524–532. doi:10.1007/s002530050855
